# Prognostic significance of coronary flow reserve assessed by MR flow measurement in the coronary sinus in patients with hypertrophic cardiomyopathy

**DOI:** 10.1186/1532-429X-15-S1-P255

**Published:** 2013-01-30

**Authors:** Yasuyuki Shiraishi, Tatsuro Ito, Masaki Ishida, Motonori Nagata, Kakuya Kitagawa, Hiroshi Nakajima, Kaoru Dohi, Masaaki Ito, Hajime Sakuma

**Affiliations:** 1 Cardiology, National hospital organization Tokyo medical center, Tokyo, Japan; 2 Radiology, Mie university hospital, Tsu, Japan; 3 Cardiology, Mie university hospital, Tsu, Japan

## Background

Previous studies using positron emission tomography demonstrated that decrease in global coronary flow reserve (CFR) predisposes to major cardiac events in patients with coronary artery disease. However, the value of MR assessment of CFR has not been clarified for predicting future cardiac events in hypertrophic cardiomyopathy (HCM). The purpose of this study was to evaluate a prognostic significance of CFR measurement in the coronary sinus in patients with HCM.

## Methods

Fifty-three patients with HCM underwent MR flow measurements in the coronary sinus during adenosine stress and in the resting state, cine MRI and late gadolinium enhanced (LGE) MRI between 2005 and 2008. CFR was calculated as the ratio of hyperemic blood flow divided by resting blood flow. After exclusion of 13 patients who had impaired LV systolic function on cine MRI (LVEF<50%, n=11) or congestive heart failure (NYHA class III-IV, n=2), 40 patients with a mean age of 62.6±10.7 years were followed for a mean duration of 4.8±1.9 years. Unfavorable outcomes were defined as cardiovascular death, unplanned cardiovascular hospital stay, and sustained ventricular arrhythmias requiring the implantation of cardioverter-defibrillator.

## Results

**Figure 1:**
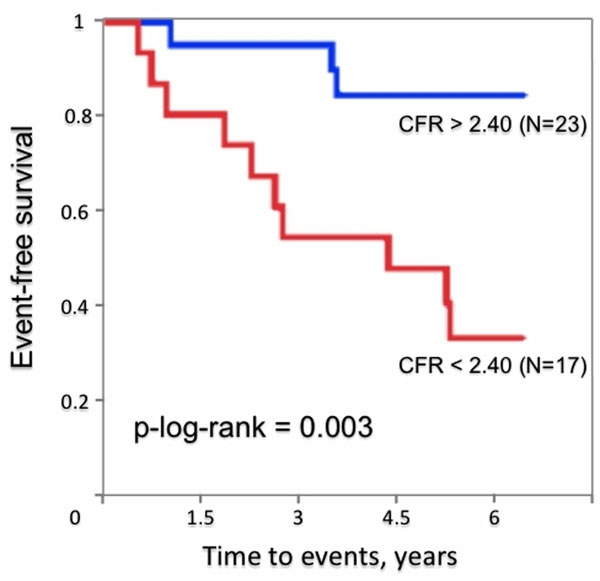
Kaplan-Meier curves showing the difference in event-free survival between patients with CFR<2.40 and CFR>2.40.

Thirteen (33%) of 40 patients had unfavorable outcomes (1 sudden cardiac death, 1 death from worsening congestive heart failure, 9 admissions for worsening heart failure, and 2 sustained ventricular arrhythmias). Reduced CFR in response to adenosine was strongly associated with unfavorable outcomes (Figure 1). Ten of 17 patients (59%) with reduced CFR of <2.4 had unfavorable outcomes, while only 3 events were observed in 23 patients with CFR of >2.40. In a stepwise multivariable Cox regression model including age, CFR, LV mass index, left atrial diameter, the presence of LGE, LV outflow tract obstruction, atrial fibrillation and familial history, reduced CFR was an independent predictor of unfavorable cardiac outcomes with a hazard ratio of 5.96 (95% CI, 1.49 to 23.80; P = 0.012).

## Conclusions

Reduced CFR assessed by MR flow measurement in the coronary sinus is a strong, independent predictor of unfavorable cardiac outcomes in HCM patients with preserved LV systolic function.

## Funding

Departmental research funding.

